# Efficacy and Safety of GLP-1 Receptor Agonists and SGLT-2 Inhibitors in the Treatment of Diabetes Mellitus and Obesity in Liver Transplant Recipients: A Systematic Review

**DOI:** 10.3390/jcm14134619

**Published:** 2025-06-30

**Authors:** Elena Garlatti Costa, Davide Bitetto, Ezio Fornasiere, Elisa Fumolo, Alberto Ferrarese, Pierluigi Toniutto

**Affiliations:** 1 Internal Medicine, Santa Maria degli Angeli Hospital, 33170 Pordenone, Italy; elenagarlatti@yahoo.it; 2 Hepatology and Liver Transplantation Unit, Azienda Sanitaria Universitaria Integrata, University of Udine, 33100 Udine, Italy; davide.bitetto@asufc.sanita.fvg.it (D.B.); ezio.fornasiere@asufc.sanita.fvg.it (E.F.); elisa.fumolo@asufc.sanita.fvg.it (E.F.); 3 Multivisceral Transplant Unit, Gastroenterology, Department of Surgery, Oncology and Gastroenterology, Padua University Hospital, 35128 Padua, Italy; alberto.ferrarese@unipd.it

**Keywords:** liver transplantation, posttransplant diabetes mellitus, glucagon-like peptide-1 receptor agonists, sodium-glucose cotransporter-2 inhibitors, obesity, graft steatosis

## Abstract

**Background/Objectives**: Glucagon-like peptide-1 receptor agonists (GLP-1RAs) and sodium-glucose cotransporter-2 inhibitors (SGLT-2Is) have significantly improved the management of diabetes mellitus (DM). In the general population, these drugs have additional benefits, such as weight loss, improvement of liver steatosis, and a cardiorenal protective effect. However, data regarding the effects of GLP-1RAs or SGLT-2Is in the treatment of posttransplant diabetes mellitus (PTDM), obesity, and their potential cardiorenal protective effects in liver transplant (LT) recipients remain limited. PTDM increases the risk of developing graft steatosis, experiencing major cardiovascular events (MACEs), and developing chronic kidney disease and reduces long-term survival in LT recipients. The aim of this systematic review was to evaluate the efficacy and safety of GLP-1RAs and SGLT-2Is in the treatment of PTDM in LT recipients. **Methods:** Twelve retrospective studies (five specifically conducted in LT recipients and seven in mixed solid organ transplant cohorts, including LT recipients) that collectively enrolled 402 LT recipients treated with GLP-1RAs and/or SGLT-2Is for PTDM were selected. **Results:** GLP-1Ras and SGLT-2Is reduced serum glycated hemoglobin levels, body weight, and insulin requirements in LT recipients. Some studies reported benefits in reducing graft steatosis, improving renal function, and in reducing the occurrence of MACEs. Common adverse events included gastrointestinal symptoms, which rarely required treatment discontinuation. **Conclusions:** GLP-1RAs and SGLT-2Is represent promising treatment options for PTDM in LT recipients, offering metabolic benefits with manageable side effects. However, further prospective studies are needed to establish the long-term safety and efficacy, as well as the favorable impact on patient survival, of these drugs in LT recipients.

## 1. Introduction

The long-term morbidity and mortality of liver transplant (LT) recipients are still strongly affected by the high incidence of malignancies and metabolic disorders, including cardiovascular disease, dyslipidemia, and hypertension, which are common among LT recipients [1,2]. These features are likely explained by the fact that nearly half of the LT recipients developed post-LT metabolic syndrome (MS), defined by the presence of three of the five criteria on the basis of the National Cholesterol Education Program Adult Treatment Panel III [1,2,3]. MS is further aggravated by excessive weight gain and the development or persistence of type 2 diabetes mellitus (DM) [4].

The pathogenesis of type 2 DM in LT recipients is multifactorial. In addition to the classic risk factors for DM recognized in the general population, such as older age, obesity, and family history of DM, LT recipients presented peculiar factors that further increase the risk of DM development. They include the presence of systemic chronic inflammation due to recurrent infections and, more importantly, the use of immunosuppressive drugs [5,6]. Glucocorticoids have been correlated with an elevated risk of DM, and calcineurin inhibitors (tacrolimus and cyclosporine) have been shown to exert a diabetogenic effect through increased insulin resistance and decreased insulin secretion [6,7,8].

The prevalence of type 2 DM in LT candidates seems to be influenced by, in addition to the IS agents, the etiology of liver disease. The prevalence of type 2 DM decreased from 49.0–72.9% in patients with metabolic dysfunction-associated steatotic liver disease (MASLD) to 24.0–24.5% in those infected with hepatitis C virus (HCV) and to 11.0–52.0% in patients with alcoholic liver disease [9,10].

The term posttransplant diabetes mellitus (PTDM) is used for every patient with newly diagnosed DM after LT irrespective of whether DM was present yet undetected before LT [5]. The incidence of PTDM at 1 year ranges from 10.8% to 33%, with an annual incidence of 3.3–30.8% [7]. PTDM is diagnosed on the basis of any of the following criteria: (a) fasting glucose > 126 mg/dL (7 mmol/L) on at least two occasions, (b) symptomatic status with random glucose > 200 mg/dL (11.1 mmol/L), (c) 2 h glucose levels after a 75 g oral glucose tolerance test (OGTT) > 200 mg/dL (11.1 mmol/L), and (d) hemoglobin A1c (HbA1c) > 6.5% once stable immunosuppression has been obtained [1,2,3,4,5,6,7,8].

Given the associations of PTDM status with increased risk of de novo or recurrent MASLD, infections, chronic kidney disease, major cardiovascular events (MACEs), and de novo malignancies, which are responsible for worse patient and graft survival [7], optimal management of this disease is of paramount importance.

Many of the pharmacological treatments for PTDM present specific barriers. Insulin use is associated with compliance, dose adjustment, and weight gain issues; metformin can cause lactic acidosis and is contraindicated for patients with severe renal impairment. Sulfonylureas are associated with a high risk of hypoglycemia and weight gain. Pioglitazone increases the risk of fluid retention and can decrease bone mineral density, whereas dipeptidyl peptidase-4 (DPP-4) inhibitors and acarbose have modest effects on glucose control [9].

Glucagon-like peptide-1 receptor agonists (GLP-1RAs) and sodium-glucose cotransporter-2 inhibitors (SGLT-2Is) have recently revolutionized the therapeutic armamentarium for patients with type 2 DM, since these drugs at the same time significantly improved glycemic control and induced protective effects on heart and kidney function [11,12]. These beneficial effects have been largely confirmed in the non-LT diabetic population [11,12,13] and in other specific clinical settings, such as patients with obesity [14] and heart diseases [15] and kidney transplant recipients [16]. GLP-1RAs mimic the physiological effects of GLP-1 by binding to the GLP-1 receptor (GLP-1R), which is expressed on pancreatic α-cells, β-cells, and δ-cells [17]. GLP-1R activation effect produced by the binding with GLP-1RAs promotes a significant effect on glycemic control in patients with type 2 DM through a several mechanisms, including stimulation of insulin secretion, inhibition of glucagon secretion, protection of β-cells from apoptosis, slowing gastric emptying, and increasing satiety [18]. In addition, semaglutide [19] and tirzepatide [20], which are the GLP-1RAs more often used, have shown an important clinical benefit in reducing liver steatosis and/or fibrosis in patients with MASLD.

SGLT-2Is are a class of antidiabetic drugs originally derived from a phenolic glucoside-denominated phlorizin. This molecule is an unspecific inhibitor of the two isoforms (SGLT-1 and SGLT-2) of SGLTs, which are mainly expressed in the intestine and kidney [21]. The more recent SGLT-2Is derivates are known as glifozins, and their use promoted the normalization of plasma glucose levels by means of development of glucosuria and high diuresis. In addition, glifozins improved insulin sensitivity and pancreatic β-cell function in animal models of DM, MS, and MASLD [22,23]. SGLT-2Is are now considered the first-line treatments for T2DM and related conditions for their insulin-independent glucose-lowering mechanism, weight-reducing effects, and improvement of kidney and cardiac function [13].

Despite the expected beneficial effects and the strong rationale for using GLP-1RAs and SGLT-2Is in treating PTDM, data on their efficacy and safety in LT recipients are still limited. Therefore, the aim of this systematic review was to evaluate the efficacy and safety of GLP-1RAs and SGLT-2Is in treating PTDM and their potential effects on weight gain and the occurrence of MACEs after LT. Furthermore, several issues that remain challenging regarding the use of these drugs for LT recipients have been highlighted.

## 2. Materials and Methods

### 2.1. Literature Search Strategy

An electronic, systematic, and comprehensive literature review was conducted and reported following the PRISMA 2020 guidelines and AMSTAR 2 (Assessing the Methodological Quality of Systematic Reviews) guidelines [24]. The literature in the Medline (through PubMed, EMBASE, Cochrane Library, ScienceDirect, and SpringerLink) databases was searched from January 2019 to April 2025. References from the included studies were also checked to identify any additional relevant papers. The following word/MESH terms were used: “(Liver transplantation, diabetes, post-transplant diabetes, GLP-1 receptor agonists, SGLT-2 inhibitors) and filters (adult, +18 years, and humans)”. The study protocol was registered on PROSPERO (ID: CRD420251039799).

### 2.2. Selection Process

All the identified records were deduplicated by three authors (E.G.C., A.F. and P.T.) using Rayyan (http://rayyan.qcri.org). A further check for the absence of duplicates was done by manually re-checking the individual selected papers. After deduplication, the remaining titles and abstracts were screened independently by the same authors using Rayyan to identify potentially eligible studies. Any disagreement over eligibility was resolved by discussion with the remaining authors (D.B., E.F. (Ezio Fornasiere) and E.F. (Elisa Fumolo)). The full texts of the selected studies were retrieved and independently assessed for eligibility by three authors (E.G.C., A.F. and P.T.), and any disagreements were resolved by discussion with the remaining authors (D.B., E.F. (Ezio Fornasiere) and E.F. (Elisa Fumolo)).

The risk of material bias was assessed using the Risk of Bias In Non-randomized Studies—of interventions, Version 2 (ROBINS-I V2) tool [25]. The assessment considered seven domains: risk of bias due to confounding, in classification of interventions, in selection of participants into the study, due to deviations from intended interventions, due to missing data, arising from measurement of outcome, and in selection of the reported results. For each study, the risk of bias was ranked as low, moderate, or high.

### 2.3. Eligibility Criteria

Randomized controlled trials (RCTs) and nonrandomized studies of interventions, including prospective and retrospective studies, assessing the effects of GLP-1RAs and/or SGLT-2Is in treating PTDM, obesity and/or MS, and graft steatosis after LT in adult patients (aged 18 years and older), were considered eligible for the analysis. Non-English papers, case reports, abstracts, letters, and studies not involving humans were excluded. Studies that included solid organ transplantation, including LT, were included but considered separately in this review.

## 3. Results

### 3.1. Description of the Included Studies

**Figure 1:**
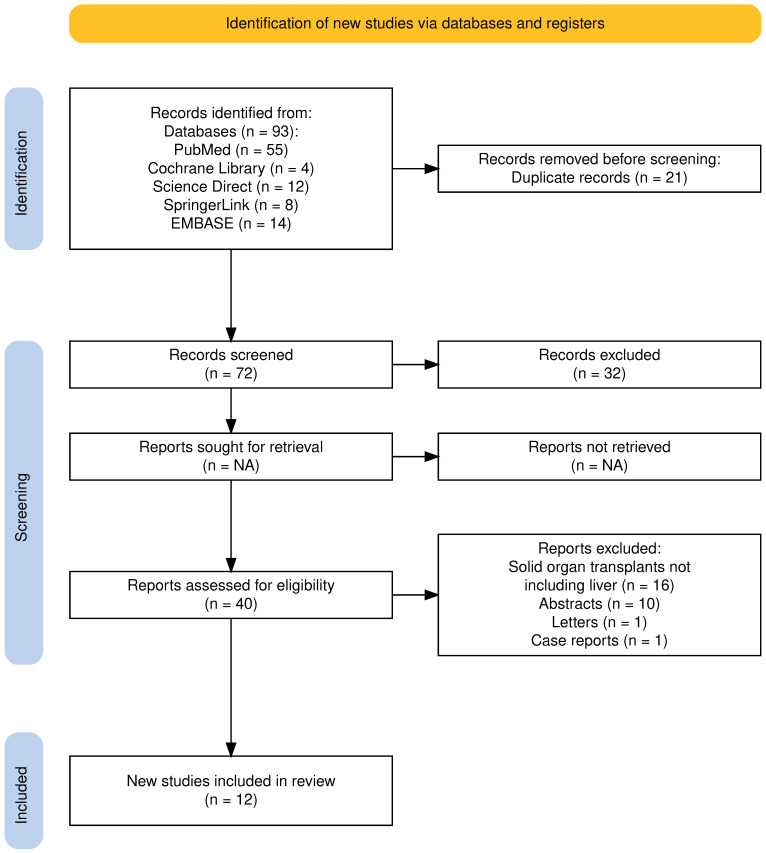
PRISMA 2020 flow diagram for the proposed systematic review, which included searches of databases and registers only. Adapted from: Page MJ, McKenzie JE, Bossuyt PM, Boutron I, Hoffmann TC, Mulrow CD, et al. The PRISMA 2020 statement: an updated guideline for reporting systematic reviews. BMJ 2021;372:71. Atthota et al. [26]; Zheng et al. [27]; Yakubu et al. [28]; Richardson et al. [29]; Chow et al. [30].

The electronic database search yielded 93 studies. Before screening, 21 studies were removed as duplicates, and 32 were removed since they were not pertinent to the aim of the review. Among the 40 studies assessed for potential eligibility, 16 were excluded because they involved solid organ transplants not including the liver, 10 because they were published as abstracts, and 2 because they were published as letter and case reports. Thus, 12 studies were selected for this review (Figure 1).

### 3.2. Characteristics of the Studies Selected

**Figure 2:**
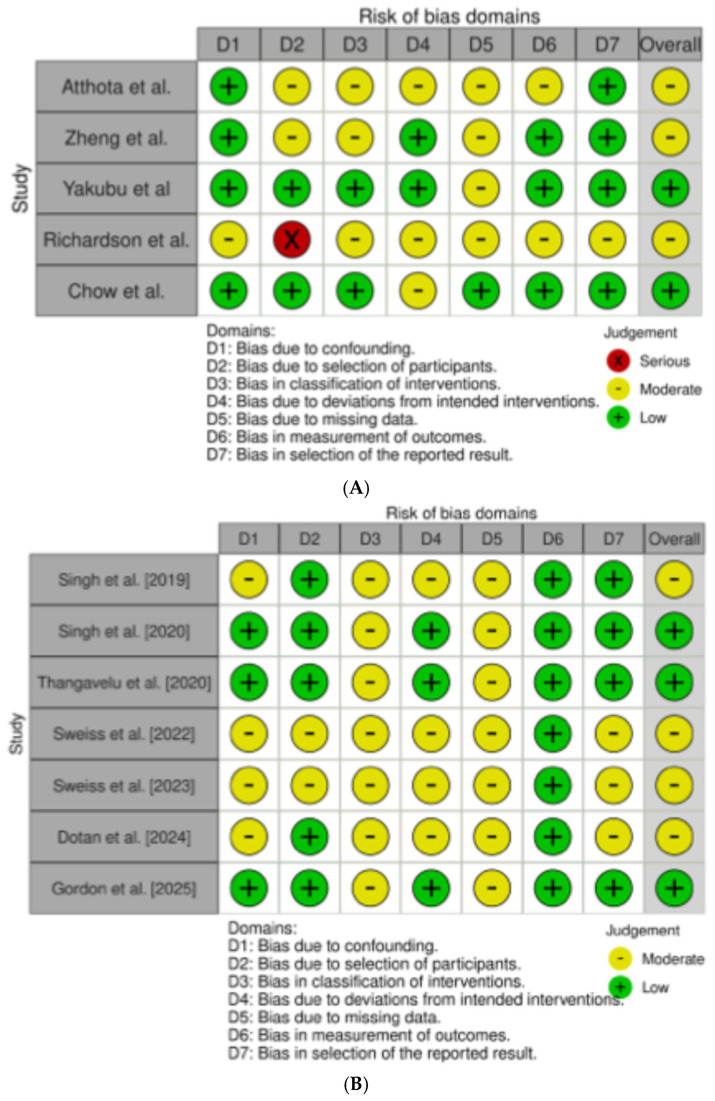
(**A**) refers to the risk of bias assessment for the studies enrolling only liver transplant (LT) recipients [26,27,28,29,30]; (**B**) refers to the risk of bias assessment for the studies enrolling solid organ transplant [31,32,33,34,35,36,37].

Among the 12 studies evaluating the impact of GLP-1RAs and SGLT-2Is in treating PTDM, weight gain, and MS in LT recipients [26,27,28,29,30,31,32,33,34,35,36,37], 5 were performed specifically in LT recipients [26,27,28,29,30], and 7 were in series of solid organ transplant recipients, in which LT or combined solid organ/LT recipients were nevertheless included [31,32,33,34,35,36,37]. All the studies selected presented a retrospective design. The overall quality of the studies was judged to have some concerns. In those specifically evaluating LT recipients, the overall risk of bias ranked as low and moderate in two [28,29,30] and three [26,27,29] studies, respectively (Figure 2A).

In the seven studies performed in solid organ transplants, including LT recipients, the overall risk of bias ranked as low and moderate in three [32,33,37] and four [31,34,35,36] studies, respectively (Figure 2B).

### 3.3. Results of the Studies Conducted Specifically in LT Recipients

In the five studies conducted specifically in LT recipients, 287 patients were treated with GLP-1RAs and/or SGLT-2Is (Table 1).

In three studies, the main indication for using GLP-1RAs and/or SGLT-2Is was PTDM, whereas in the remaining two studies, the main indications were obesity and/or overweightness, but PTDM was the main comorbidity.

In the study by Atthota et al. [26], 37 LT recipients were enrolled, and 29 and 8 were treated with GLP-1RAs and SGLT-2Is, respectively, with a median follow-up of 427 days. PTDM, which was already present before LT in 84% of the patients, was the most common indication for treatment, followed by obesity. Furthermore, in 21 patients, graft steatosis was documented before antidiabetic treatment. Treatment with GLP-1RAs and/or SGLT-2Is was associated with a significant decrease in body mass index (BMI), HbA1c, and the number of insulin units needed. Among the 10/21 patients in whom the change in graft steatosis grade was evaluated, 5 patients improved, 4 worsened, and 1 remained unchanged. For five (13.5%) patients, treatment was interrupted due to gastrointestinal discomfort or unstable renal function.

A larger number of LT recipients were evaluated in the study by Zheng et al. [27], who retrospectively enrolled 145 LT recipients presenting with PTDM. Among them, 46, 87, and 12 patients received GLP-1RAs, SGLT-2Is, or the combination of both, respectively. Several metabolic and biochemical parameters were collected for up to 12 months after starting treatment and were compared to those recorded in 217 LT recipients treated for PTDM with DPP-4 inhibitors. Both GLP-1RA treatment and combination therapy significantly decreased the mean serum HbA1c level. In addition, the effect of combination therapy remained significant after adjustment for DPP-4 inhibitor treatment. A decrease in body weight and BMI was significantly associated with the use of GLP-1RAs or combination therapy. Interestingly, compared with those receiving monotherapy or DPP-4 inhibitors, patients receiving combination therapy showed a nonsignificant improvement in the estimated glomerular filtration rate (eGFR), particularly after 3 to 6 months of treatment. A slight and transient decrease in tacrolimus serum levels was observed in SGLT-2I users, which was significantly less pronounced than that in DPP-4 inhibitor users. Regarding the occurrence of adverse events, two cases (1.4%) of T-cell-mediated rejection (TCMR) were observed: one in the combination treatment group and one in a patient receiving SGLT-2Is alone.

The effects of GLP-1RA treatment on graft steatosis development, body weight changes, and glycemic control were compared to those of insulin in a 1:1 matched series of 38 LT recipients by Yakubu et al. [28] in a retrospective single-center study. The authors demonstrated that treatment with GLP-1RAs induced better glycemic control and an 8% decrease in body weight, compared with the 10% increase in body weight observed in insulin-treated patients. Furthermore, patients receiving GLP-1RAs were less likely to develop graft steatosis. Similarly to the previously mentioned studies, nonrelevant adverse events related to GLP-1RA use were observed.

The favorable effects of GLP-1RA use on body weight in LT recipients who developed obesity was confirmed in a small retrospective study performed by Richardson et al. [29]. In this study, 29 obese LT recipients were treated with GLP-1RAs and 15 were not. Compared with the latter group, the former group experienced a greater percentage of weight loss from baseline (7.87% vs. 4.24%) at 1 year. The incidence of MACEs during the study period was <10%, and no TCMR episodes were observed. Three (6.9%) recipients treated with GLP-1RAs reported nausea and/or vomiting, which did not lead to medication discontinuation.

Similar results were obtained by Chow et al. [30], who treated 23 overweight/obese LT recipients (70% with PTDM) with semaglutide at weekly doses ranging from 0.20 mg to 2 mg. After a mean follow-up of 17 months, the mean body weight was significantly lower (87 kg vs. 92 kg) than that recorded before semaglutide initiation. The semaglutide-related side effects were abdominal discomfort, nausea, and diarrhea in approximately 30% of patients, but no drug dose reductions or discontinuations were needed.

### 3.4. Results of the Studies Conducted in Solid Organ Transplant Recipients, Including LT

In the seven studies enrolling solid organ transplant recipients, 115 LT recipients were treated with GLP-1RAs, SGLT-2Is, or a combination of both (Table 2).

These studies yielded results comparable to those reported in studies specifically enrolling LT recipients. The study by Singh et al. [31] is currently the largest single-center study in which 20 solid organ transplant recipients with PTDM were treated with dulaglutide. In this study, 63 solid organ transplant recipients (10 LT recipients) received dulaglutide at a dose of 0.75 mg (maximum 1.5 mg) weekly. The primary endpoints were changes in body weight, BMI, insulin requirements, the occurrence of MACEs, graft survival, and all-cause mortality. The secondary endpoints were HbA1c, serum creatinine, eGFR, and liver function test changes. The main results were that at 6, 12, and 24 months after dulaglutide initiation, significant weight loss and a decrease in BMI were observed. Moreover, 47% of patients reduced their daily insulin requirement, and 13% discontinued all other antidiabetic medications. A nonsignificant improvement in both the eGFR and creatinine levels was observed during the follow-up period. None of the patients showed an increase in serum transaminases. After 6 months of dulaglutide treatment, HbA1c was reduced in 65% of patients, increased in 24%, and remained the same in 11% of patients. No MACEs were observed. With respect to dulaglutide safety, 6.3% of patients developed hypoglycemia that did not require hospitalization, and 1.5–3% of patients reported no severe nausea, vomiting, diarrhea, or abdominal pain. The same author in a subsequent study [32] compared these 63 patients (10 LT recipients) who received dulaglutide with 25 solid organ transplant recipients (1 LT recipient) who received liraglutide at a subcutaneous daily dose ranging from 0.6 mg to 1.8 mg for treating PTDM. The primary and secondary endpoints were the same as those previously reported. Both body weight and BMI decreased more significantly during the study period in patients receiving dulaglutide than in those receiving liraglutide. Similarly, the percentage reduction in the insulin requirement was significantly greater in the dulaglutide-treated group than in the liraglutide-treated group. At the end of follow-up, a 10% reduction in the creatinine level and a 15% increase in the eGFR were observed in the dulaglutide group, whereas in the liraglutide group, the creatinine level increased by 7% from baseline, although in all groups, the immunosuppressive regimen remained stable. Approximately 14% of patients in the dulaglutide group and 8% in the liraglutide group discontinued all antidiabetic medications and were maintained only on GLP-1RAs. There was a nonsignificant trend toward an increasing rate of cardiovascular morbidity in the liraglutide-treated group. The development of hypoglycemia episodes and gastrointestinal discomfort was greater in the liraglutide group than in the dulaglutide group, and interestingly, four patients developed cholelithiasis.

In a small series of 19 solid organ transplant (7 LT) recipients, Thangavelu et al. [33] showed that the use of GLP-1RAs for treating PTDM was associated with a significant decrease in body weight and HbA1c serum levels without significant drug-drug interactions with immunosuppressive agents.

These results were confirmed in the more recent largest study evaluating the efficacy of GLP-1RAs in treating PTDM after solid organ transplantation performed by Sweiss et al. [34]. This study included 118 solid organ transplant (23 LT) recipients who received GLP-1RAs (liraglutide 31%, dulaglutide 38%, semaglutide 27%, exenatide 4%) for at least 3 months to treat PTDM, which was present before transplant in 68% of the patients. Fasting glucose, body weight, BMI, serum creatinine, eGFR, and the median dose of insulin required significantly decreased from baseline to the 3- to 12-month nadir. An HbA1c level < 7.5% at follow-up was obtained for 45.8% of the patients. Nine (7.6%) patients discontinued GLP-1RA use after a minimum of 3 months of therapy, but only two discontinued it because of major adverse effects. Three patients (2.5%) were hospitalized: one for TCMR, one for graft dysfunction, and one for increased serum transaminase levels. The same author [35] evaluated the 12-month efficacy of SGLT-2Is alone or in combination with GLP-1RAs in treating PTDM in a cohort of 38 solid organ (26 LT and 1 combined liver–kidney transplant) recipients. The authors confirmed that, after initiating therapy with an SGLT-2I, a significant reduction in HbA1c and fasting blood glucose compared with pre-SGLT-2I initiation was observed. Furthermore, a significant reduction in body weight and BMI was also observed. The most common adverse effect was hypoglycemia, which led to drug discontinuation in two patients. In the study by Dotan et al. [36], 338 solid organ transplant recipients (28 LT recipients) with PTDM were enrolled. Patients were divided into two comparable arms (including 14 LT recipients per arm). One group received GLP-1Ras, and the other group served as control. The primary outcome was a composite of MACEs, and the secondary outcomes were MACE or peripheral vascular disease and all-cause mortality. The study showed that solid organ transplant recipients with PTDM who received GLP1-RAs had lower risks for MACE and all-cause mortality.

Gordon et al. [37] retrospectively evaluated 70 solid organ transplant recipients (10 LT recipients) who presented with PTDM. Patients were matched 1:1 in two groups: those who received a GLP-1-RA-containing insulin regimen or a regimen containing insulin only, initiated within 12 months of transplant. The aim of this study was to evaluate HbA1c levels after 12 months of antidiabetic treatment. The main results of the study were that insulin use decreased to 69% in the GLP-1RA group, whereas 94% of the subjects in the insulin-only group remained on insulin. Furthermore, there were 7.2 fewer injections per week in the GLP-1RA group than in the insulin group. Regarding side effects, as expected, a significantly greater incidence of nausea was recorded in the GLP-1RA group than in the insulin-only group. Surprisingly, this study revealed a greater incidence of TCMR in GLP-1RA-treated patients, who reached 29%, than in insulin-only patients, who reached 6%. The authors did not report an explanation of this result, although they did not attribute the higher rate of TCMR to the use of GLP-1RAs.

## 4. Conclusions and Challenging Issues

Both GLP-1RAs and SGLT-2Is seem to have efficacy and safety in treating PTDM in LT recipients, as well documented in the general population. Furthermore, the side effects of these drugs seem to be easily manageable, leading to drug discontinuation in a very small number of patients. Thus, transplant hepatologists should be encouraged to use this type of drug more confidently in the treatment of PTDM, obesity, and graft steatosis in LT recipients.

Despite these promising results, several issues remain challenging. All the studies analyzed in this systematic review were retrospective. This represents a serious limitation in the interpretation of the results, since a few selection biases cannot be excluded. The different time intervals between LT and introduction of GLP-1 RAs and/or SGLT-2Is makes patients not homogeneous among the different studies. It is therefore possible that the efficacy and tolerability of therapies was influenced by the duration of PTDM and by the presence of concomitant organ damage associated with it. Some potentially confounding factors should be considered. These are related to the type of antidiabetic therapy used in the studies, which was not standardized across all patients enrolled. The choice between GLP-1RAs and/or SGLT-2Is was not made on the basis of a uniform criterion or following the updated clinical guidelines, which have been designed for the general population [38,39]. Most of the studies focused on the use of GLP-1RAs. This could be explained by the existence of barriers in using SGLT-2Is in patients with renal impairment related to the use of immunosuppressive drugs. Furthermore, the risk to induce more frequently urinary tract infections in LT recipients could be perceived as higher than the general population [40]. The short follow-up period of these studies induces to consider with caution the beneficial effect of combined GLP-1RA and SGLT-2I treatment in reducing the occurrence of MACEs, as observed in the non-LT population [11]. However, the better glycemic control and weight loss observed in LT recipients treated with GLP-1RAs and/or SGLT-2Is could suggest that the cardiovascular benefits of these drugs should be confirmed even in this population. Some caveats remain regarding the contradictory results obtained in the studies regarding the increase in TCMR episodes observed in patients receiving GLP-1RAs. A possible explanation for this observation, however unproven, could be that the reduction in gastric emptying associated with the intake of these drugs [41] may alter the absorption of immunosuppressive agents, causing a reduction in their plasma levels and an increased risk of developing TCMR episodes. Since the TCMR remains a cause of graft loss in LT patients [42], the potential impact of GLP-1RAs in enhancing the risk for developing of TCMR must be carefully evaluated in prospective studies. The potential increased risk of developing biliary stones related to GLP-1RA use should also be carefully evaluated in prospective studies, since anastomotic or ischemic biliary strictures are common after LT [2]. Finally, the short-term follow-up of these studies cannot confirm whether the beneficial clinical effects of GLP-1RA and/or SGLT-2I treatment could improve the long-term survival of LT recipients. To demonstrate this strong clinical benefit, well-designed prospective studies are urgently needed.

## Figures and Tables

**Table 1:** Studies evaluating the efficacy and safety of glucagon-like peptide-1 receptor agonists (GLP-1RAs) and sodium-glucose cotransporter-2 inhibitors (**SGLT-2**Is in liver transplant (LT) recipients.

Author	Study Type	Patients (N.)	PTDM	Obesity	Drug Type	Time from LT to Tx	Comparator	F-Up Time	Primary Endpoint	Secondary Endpoint	Main Results
Atthota et al. [26]	Single-center retrospective, not randomized	37	Yes	Yes	GLP-1RAs (29) SGLT-2Is (8)	1243 days	None	427 days	Body weight, HbA1c	Graft steatosis Use of insulin Safety	Hb A1c, BMI, and insulin dose decreased No safety concerns
Zheng et al. [27]	Single-center retrospective, not randomized	145	Yes	No	GLP-1RAs (46) SGLT-2Is (87) Both (12)	90 days	DPP-4 inhibitor	12 months	HbA1c BMI eGFR AST ALT	Safety	HbA1c levels decreased at 12 months ALT and BMI decreased in GLP-1RA and combo therapy eGFR stable TCMR in 1.4%
Yakubu et al. [28]	Single-center retrospective, randomized	38	Yes	No	GLP -1 RA (38)	48 months in GLP-1RA group; 11 months in insulin group	Insulin	12 months	Metabolic effect	Safety Graft steatosis	Weight loss and HbA1c reduced at 12 months in GLP-1RA users Same LDL, HDL, TG, AST levels No MACE eGFR remained stable Lower graft steatosis in GLP-1RA users No TCMR

PTDM: posttransplant diabetes mellitus; NR: not reported; SEMA: semaglutide; DPP-4: dipeptidyl peptidase-4 inhibitors; HbA1c: glycated hemoglobin; BMI: body mass index; eGFR: estimated glomerular filtration rate; AST: aspartate aminotransferase; ALT: alanine aminotransferase; MACE: major cardiovascular events; TCMR: T-cell-mediated rejection; LDL: low-density lipoprotein; HDL: high-density lipoprotein; TG: triglyceride.

**Table 2:** Studies evaluating the efficacy and safety of glucagon-like peptide-1 receptor agonists (GLP-1)RAs and sodium-glucose cotransporter-2 inhibitors (SGLT-2Is in solid organ transplants, including liver transplant (LT) recipients.

Author	LT Recipients Included (N.)	Type of Drug Used	Main Results
Singh et al. [31]	10	GLP-1RAs	Sustained reduction in weight, BMI, and insulin requirement Gastrointestinal manifestations were rare
Singh et al. [32]	11	GLP-1RAs	Sustained reduction in weight, BMI, and insulin requirement
Thangavelu et al. [33]	7	SGLT-2Is	GLP-1RAs do not affect tacrolimus levels or transplant outcomes GLP-1RAs were effective for glycemic control and weight loss
Sweiss et al. [34]	23	GLP-1RAs	Reduction in HbA1 and weight loss The rate of adverse drug reactions was low
Sweiss et al. [35]	26	SGLT-2Is	Significant benefit on glycemic control promoting weight loss Good total safety outcomes
Dotan et al. [36]	28	GLP-1RAs	Diabetic transplant recipients who used GLP-1RAs had lower risk of experiencing MACEs and all-cause mortality
Gordon et al. [37]	10	GLP-1RAs	Insulin use decreased to 69% in the GLP-1RA group, while 94% of subjects in the insulin-only group remained on insulin. A significantly higher incidence of TCMR reported in the GLP-1RA group (29%) than that in the insulin-treated group (6%)

BMI: body mass index; HbA1: glycated hemoglobin; MACEs: major cardiovascular events; TCMR: T-cell-mediated rejection.
